# Individual surgeon mortality rates: can outliers be detected? A national utility analysis

**DOI:** 10.1136/bmjopen-2016-012471

**Published:** 2016-10-03

**Authors:** Ewen M Harrison, Thomas M Drake, Stephen O'Neill, Catherine A Shaw, O James Garden, Stephen J Wigmore

**Affiliations:** 1 Clinical Surgery, Surgical and Perioperative Health Research (SPHeRe), University of Edinburgh, Royal Infirmary of Edinburgh, Edinburgh, UK; 2 The Medical School, University of Sheffield, Sheffield, UK

**Keywords:** SURGERY, Patient safety, Patient outcome assessment, Surgeons/standards, Operative/mortailty

## Abstract

**Objectives:**

There is controversy on the proposed benefits of publishing mortality rates for individual surgeons. In some procedures, analysis at the level of an individual surgeon may lack statistical power. The aim was to determine the likelihood that variation in surgeon performance will be detected using published outcome data.

**Design:**

A national analysis surgeon-level mortality rates to calculate the level of power for the reported mortality rate across multiple surgical procedures.

**Setting:**

The UK from 2010 to 2014.

**Participants:**

Surgeons who performed colon cancer resection, oesophagectomy or gastrectomy, elective aortic aneurysm repair, hip replacement, bariatric surgery or thyroidectomy.

**Outcomes:**

The likelihood of detecting an individual with a 30-day, 90-day or in-patient mortality rate of up to 5 times the national mean or median (as available). This was represented using a novel heat-map approach.

**Results:**

Overall mortality rates for the procedures ranged from 0.07% to 4.5% and mean/median surgeon volume was between 23 and 75 cases. The national median case volume for colorectal (n=55) and upper gastrointestinal (n=23) cancer resections provides around 20% power to detect a mortality rate of 3 times the national median, while, for hip replacement, this is a rate 5 times the national average. At the mortality rates reported for thyroid (0.08%) and bariatric (0.07%) procedures, it is unlikely a surgeon would perform a sufficient number of procedures in his/her entire career to stand a good chance of detecting a mortality rate 5 times the national average.

**Conclusions:**

At present, surgeons with increased mortality rates are unlikely to be detected. Performance within an expected mortality rate range cannot be considered reliable evidence of acceptable performance. Alternative approaches should focus on commonly occurring meaningful outcome measures, with infrequent events analysed predominately at the hospital level.

**Strengths and limitations of this study**

- This national analysis of individual surgeon mortality rates identified that surgeons with increased mortality rates are unlikely to be detected.
- Stratification of procedures by risk-identified surgeons undertaking operations with low mortality risks would be unlikely to perform enough procedures in their lifetime to detect poor performance.
- Alternative measures of outcomes such as functional health status or patient satisfaction may be more suitable, detect poor performance earlier and have greater power to detect outlying surgeons or units.
- Future research and policymakers should consider implementing alternative measures alongside mortality rates that are reported at the surgeon and unit level.

## Introduction

Ensuring the highest quality of care from individuals working in health services is a priority worldwide. A desire to identify and learn from best practice is accompanied by a requirement to detect performance that falls below expectation. There has been debate within a number of countries and surgical specialties on the pros and cons of making public the results of individual surgeons,^1^ with a particular focus on mortality rates.

In June 2013, the National Health Service in England published for the first time mortality rates for individual surgeons. It was described as a major breakthrough in transparency that could help drive up standards.^2^ Concerns were raised including whether surgeons performed particular procedures frequently enough to enable those with an excess mortality to be reliably identified. A study at the time used hospital episode statistics to estimate individual surgeon volume and concluded that public reporting of surgeon outcomes could lead to false complacency among the public and surgical community.^3^ Furthermore, controversy exists at least in cardiac surgery on the extent to which the operating surgeon can modify the risk of death for an individual patient.^4^

Despite concerns about the use of mortality as a marker of individual surgeon performance,^5^ it continues to be used.^6^ Acceptable performance exists within an expected range, which can be defined indifferent ways.^7^ It is common to compare an individual surgeon's performance with that of population and consider a mortality rate of >2 SDs from national mean/median to indicate divergent practice. This definition shows that there is an expected false-positive rate of 5%. For example, a surgeon with a mortality rate equal to that of the national mean or median has a 2.5% chance of falling above the 95% control limit for acceptable performance by sampling error alone.

The chances of detecting a surgeon with a mortality rate that is actually worse than the national average is a question of statistical power. As caseload increases, so does the power to detect divergent practice. In this context, the statistical power can be considered the likelihood of detecting an individual surgeon with a mortality rate in excess of the national average: a power of 100% would detect every surgeon with a mortality rate in excess of the national mean/median, while 50% power would detect half of these surgeons.

The aim of this study was to examine the available mortality data for individual surgeons and to determine the likelihood that variation in surgeon performance will be detected.

## Methods

### Data

National mean or median mortality rates, together with case volume data for the UK surgeons, were retrieved for six procedures on 1 June 2015.^8^ The mean or the median mortality rate was used as reported in the data. The primary outcome of 30-day, 90-day or in-patient mortality was used as available. The following procedures were included and outcomes were available follows: colon cancer resection (90-day mortality), upper gastrointestinal (GI) cancer resection (30-day and 90-day mortality), elective abdominal aortic aneurysm repair (30-day mortality), hip replacement surgery (90-day mortality), bariatric surgery (in-hospital mortality) and thyroidectomy (in-hospital mortality). The available data covered procedures that were performed from 2010 to 2014.

### Analysis

Mortality and procedural case volume data reported for surgeons were characterised. Data across all available years (2010–2014) were aggregated for each procedure and mapped using a heat-map approach. This heat-map was used to show the likelihood of detecting an individual surgeon with a mortality rate of two, three, four and five times the national mean/median, that is, the proportion above an exact 95% binomial control limit determined using the Wilson method.^9^ The mean/median case volume and range for surgeons reported in national data are superimposed on to the plots. The proportion of individuals above the 95% control limit was represented in blue, with darker colour indicating a higher proportion. In other words, the colour represents the statistical power of detecting a mortality rate 2–5 times the average. The black vertical lines represent the mean or median average mortality rate (dependent on the measure reported in the accessed data). The red vertical lines represent the minimum and maximum number of cases performed for the given data set, with the x-axis indicating the number of procedures per surgeon.

Data were analysed using R V.3.2.1 (R Foundation for Statistical Computing, Austria) using the packages ‘binom’ and ‘ggplot2’.

## Results

### Reported data

There was variation in the outcome measures reported across outcome data. For upper GI cancer resection, 30-day and 90-day mortality were reported. Whereas, for other procedures, only 30-day, 90-day or in-patient mortality were published. Different measures of central tendency were reported for case volume and mortality rate, with some audits reporting the mean average and others reporting the median.

### Mortality rates and procedure volume

The higher risk procedures included upper GI cancer resection, colorectal cancer resection and elective aortic aneurysm repair (mortality rate 2.2–4.5%, table 1). Lower risk procedures were bariatric surgery, thyroidectomy and hip replacement (mortality rate 0.07–0.4%, table 1). The national average surgeon volume for the six procedures was between 23 and 75 cases.

**Table 1:** Summary of surgeons and procedures

Procedural risk	Procedure	Surgeons (n)	Mortality measure	Overall mortality rate (%)	Average case volume per surgeon, n (range)
High	Upper GI cancer resection	195	30-day	2.40	23 (10–81)*
	Upper GI cancer resection	195	90-day	4.50	23 (10–81)*
	Colon cancer resection	742	90-day	3.00	55 (3–237)*
	Elective abdominal aortic aneurysm repair	417	30-day	2.20	32 (1–237)*
Low	Hip replacement	1625	90-day	0.40	48 (1–529) †
	Bariatric surgery	124	In-hospital	0.07	75 (5–160) †
	Thyroidectomy	175	In-hospital	0.08	69 (10–210)*

*Central tendency reported as median.

†Central tendency reported as mean.

GI, gastrointestinal.

### Higher risk procedures

In published outcomes for colorectal cancer resection, the median number of procedures submitted by individual surgeons for the 3-year period analysed was 55 (range 3–237; figure 1). With a median national 90-day mortality rate of 3.0%, the national median of 55 cases provides around 20% power to detect a mortality rate three times the national median. Put another way, for this 3-year period, around 20 out of 100 individuals with an actual mortality rate of 9% would appear above a 95% control limit. The case volume per surgeon over 3-years would have to be over 200 procedures to have 90% power of detecting a surgeon with a 90-day mortality rate three times the national median.

**Figure 1:**
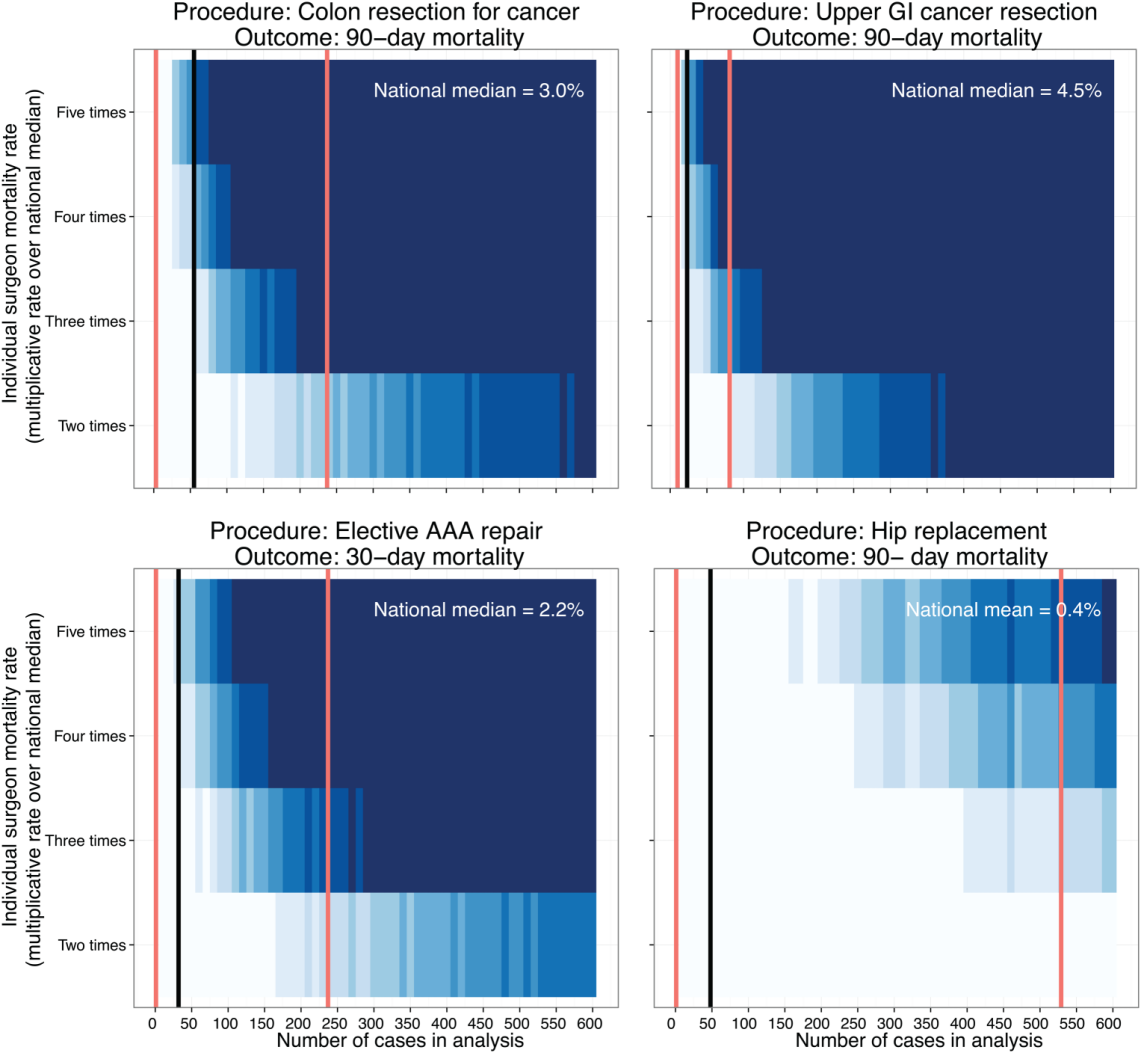
Proportion of a group of surgeons with mortality rates higher than the national average predicted to be detected (statistical power). Mortality data and procedural case volume reported for surgeons were retrieved from NHS Choices website.^8^ The heat-map shows the likelihood of detecting an individual surgeon with a mortality rate in excess of the national mean/median (ie, the proportion above an exact 95% control limit (Wilson method) on a funnel plot). The rate of the mortality measure above the national mean/median on the y-axis is plotted against case volume on the x-axis. The mean or median case volume (black line) and range (red lines) of surgeons reported in these national data are also shown. For example, with colonic resection, the national median caseload of 55 provides only around 20% power to detect a mortality rate three times the national median. Two hundred cases provide >90% power. Owing to the discrete nature of these data, as expected, the gradient from left to right is not always smooth.

**Figure 1:**
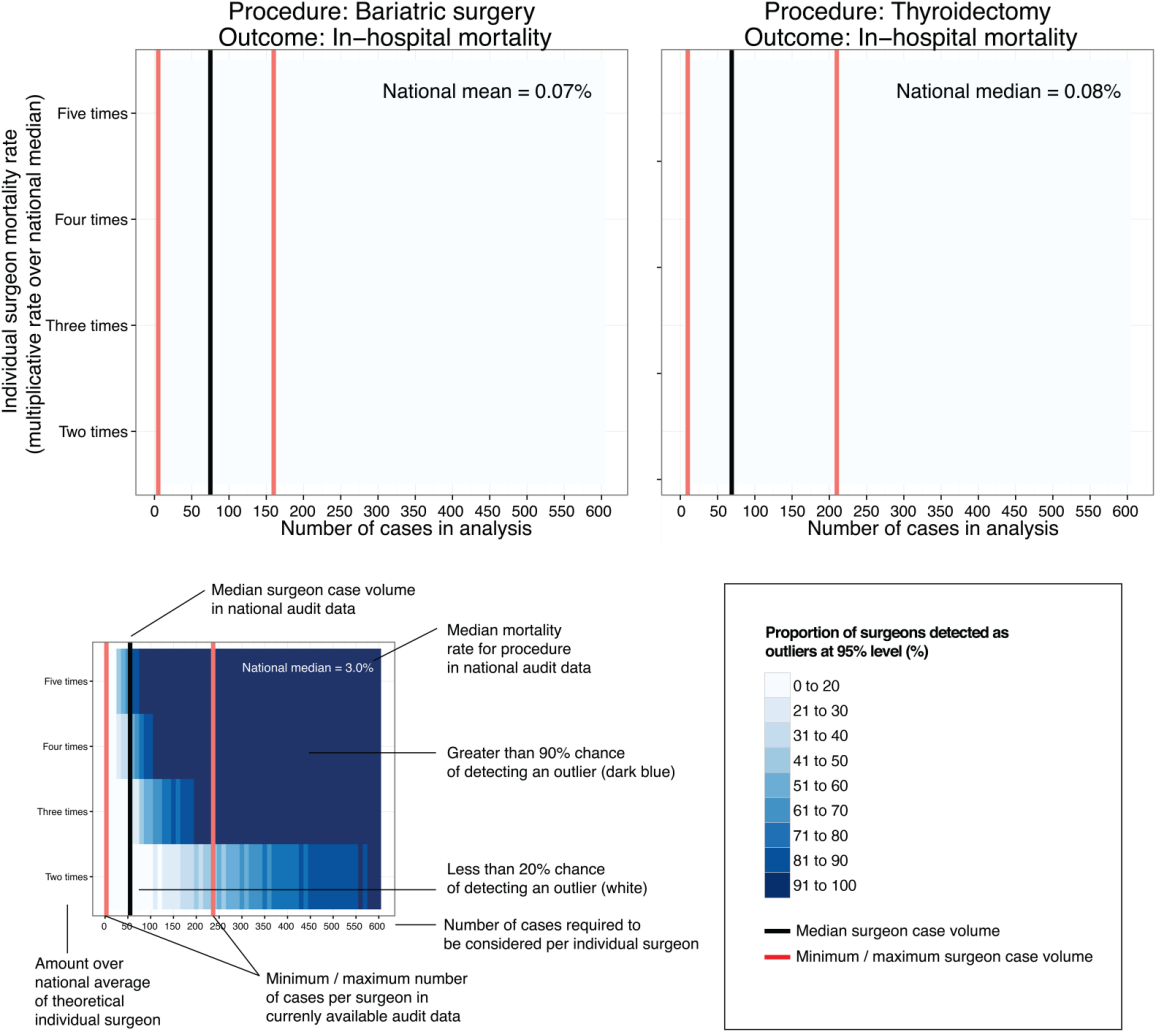
Continued

Similar findings were seen for upper GI cancer resection, where the median number of procedures submitted by individual surgeons over a 2-year period was 23 (range 10–81) (figure 1). With a median national 30-day mortality rate of 2.4%, the cases submitted provided <20% power to detect a surgeon with a mortality rate four times the national median. Likewise, a 90-day mortality rate three times the national median can only be detected with a power of <20%. A 2-year case volume of 300 cases would be required to have 80% power to detect a 90-day mortality rate two times higher than the national median.

### Lower risk procedures

For hip replacement, bariatric procedures and thyroidectomy, at the national mean or median case volumes submitted (48–75 cases per surgeon, range 1–529) fewer than 20 of 100 individuals with an actual mortality rate five times the national average would be detected. For hip replacement, an annual case volume in excess of 500 cases would be required to have 80% power to detect an individual with a mortality rate five times the national average (0.4%). For thyroidectomy and bariatric procedures, the reported national mortality rates are even lower (0.07% and 0.08%). At these rates, it is unlikely that a surgeon would perform a sufficient number of procedures in his/her entire career to stand a good chance of detecting a mortality rate five times the national average.

## Discussion

This is the first study of which we are aware to analyse the utility of national individual surgeon mortality rates. On the basis of these rates and published case volumes, surgeons with mortality rates in excess of that expected are highly unlikely to be detected. Performance within an expected mortality rate range cannot therefore be considered a reliable evidence of acceptable performance. Robust interpretation of performance data for individual surgeons clearly has important implications for patient care. In the UK, the National Clinical Audit Advisory Group has produced guidance on the detection and management of performance outliers identified through national clinical audit.^7^ When a healthcare provider is detected as a potential performance outlier, an escalating confirmatory process is embarked on which may quickly result in the suspension of a service or individual. This analysis demonstrates that, for these common procedures, mortality rates are not a robust method for detecting divergent practice. It is not surprising that the performance of all but one surgeon across all six procedures was found to be acceptable.

This study has several limitations that must be considered. Data reporting standards for individual surgeon mortality rates varied across different specialties, with some presenting the median and some the mean average. Further variability in the outcome measures reported (30-day, 90-day or in-patient mortality) made it difficult to use a uniform approach across all data sets. In future, surgeon-level surgical mortality should be standardised to consider 90-day outcomes as a sensitive measure of mortality. We addressed this by analysing each procedure individually and ensuring the relevant measures of central tendency were clearly specified for each procedure. A further strength of this study is the national population of surgeons included, thus reducing the risk of selection or attrition bias from unobserved outcomes.

The recent push for the publication of individual surgeon outcomes underpins a specific public interest in safer surgery. Surgeons broadly support greater transparency in the publication of outcome data, but these data should be meaningful and accompanied by appropriate presentation and interpretation. An example of the difficulties in delivering this is the recent publication of complication rates for 17 000 US surgeons.^10^ ProPublica, an independent, non-profit investigative journalism organisation, used Medicare data to perform the largest public analysis of surgeon-level complications rates. Although initiatives of this type may be welcomed, the analysis and presentation of the data has been criticised and described as misleading.^11^ In particular, the number of cases available per surgeon is low and the power of any analysis at the individual level limited. Concerns have previously been raised about using mortality in this manner,^5^ and as has been shown in the current analysis, when case volumes are low and outcomes are infrequent, it is not possible to perform a meaningful analysis at the individual surgeon level.

One obvious solution to better estimate individual outcomes is to increase the case volume considered. Case ascertainment in an national audit is important but not always well defined. Average case volumes analysed in the present study are lower than the predicted by hospital episode statistics. It has long been debated the extent to which minimum volume standards should be set for surgeons performing a given procedure. Volume–outcome relationships are well described for many procedures and while efforts have been made to concentrate specialist surgical expertise, policymakers have stopped short at enforcing volume targets. Three leading hospital systems in the USA—Dartmouth-Hitchcock Medical Center, Johns Hopkins Medicine and the University of Michigan—have recently sought to introduce minimum volume standards for 10 surgical procedures.^12^ Yet even the minimum numbers suggested here would be insufficient to make mortality rates a useful marker of individual outcome, for example, pancreas resection, 5 per year; hip replacement, 25 per year. In future, the measurement of mortality may become an increasingly poor means of discriminating surgeon performance if postoperative death rates continue a downward trend.^13^ ^14^

Our analysis would strongly suggest an alternative approach, focus measurement on commonly occurring meaningful outcomes, analyse infrequent events predominately at the hospital level and use prospective continuous methods rather than retrospective snapshots. The more frequently an outcome measure occurs, the greater its utility in differentiating better care. An outcome is only meaningful if it directly measures or acts as a surrogate for factors explicitly relevant to a patient. Specific patient-centred outcomes—such as patient satisfaction, functional health status or other measures of health-related quality of life—are ideal yet far from routine; we are not aware of any comprehensive system reporting at the level of the individual surgeon. The appropriateness of more traditional measure depends on the balance between baseline risk and case volume.^15^ Measures of structure (eg, volume), process (eg, venous thromboembolism prophylaxis, antibiotic prophylaxis or nutritional support) or direct outcomes (eg, well-defined complication rates or readmission rates) can be useful when deployed for the appropriate procedure.

When measuring these wide-ranging outcomes, caution must be exercised when performing an analysis at the level of an individual practitioner. It is the performance of the healthcare system as a whole that is likely to affect, for instance, overall patient satisfaction rather than that only of the surgeon. Undoubtedly, for less frequently performed surgical procedures, analysis at the level of the hospital will be more meaningful—it has even been suggested that for procedures of low risk or caseload, efforts would be better targeted on the measurement of other procedures.^15^

Future research and policymakers should focus on implementing measures that can detect deterioration in performance in a sensitive manner, to enable early detection and accurate discrimination between surgeons, units or hospitals. If the performance of a hospital begins to deviate over time, an early in-depth review may identify modifiable causes. It may be difficult to identify a poorly performing individual if he/she is compensated for by colleagues, but this may be better addressed by regular internal appraisal and multisource feedback. Assessing performance retrospectively may lead to delays in identifying divergent practice. Continuous monitoring of outcomes from consecutive cases has instinctive appeal and practical advantages. Graphical measures such as the variable life-adjusted display (VLAD) (or expected-observed cumulative sum (CuSum) plot) were established to display differences between observed and expected mortality in cardiac surgery.^16^ This continuous approach is, however, at the expense of more frequent reviews of surgeons performing in the expected range, but such reflective practice should be encouraged and become a more common part of everyday practice. It may be instructive to consider how often surgeons performing well should expect to trigger a false positive. For surgeons performing below expectation, these early alerts will potentially improve patient safety when attention or reskilling may lead to improvement.^17^
